# Potential for spatial coexistence of a transboundary migratory species and wind energy development

**DOI:** 10.1038/s41598-024-66490-3

**Published:** 2024-07-24

**Authors:** Ta-Ken Huang, Xiao Feng, Jonathan J. Derbridge, Kaitlin Libby, Jay E. Diffendorfer, Wayne E. Thogmartin, Gary McCracken, Rodrigo Medellin, Laura López-Hoffman

**Affiliations:** 1 Department of Water Resources and Environmental Engineering, Tamkang University, No.151, Yingzhuan Rd., Tamsui Dist., New Taipei City, 251301 Taiwan; 2 School of Natural Resources and the Environment, The University of Arizona, 1064 East Lowell Street, Tucson, AZ 85721 USA; 3 Department of Geography, Florida State University, 113 Collegiate Loop, PO Box 3062190, Tallahassee, FL USA; 4 US Geological Survey, Geosciences and Environmental Change Science Center, P.O. Box 25046, DFC, MS980, Denver, CO 80225 USA; 5 US Geological Survey, Upper Midwest Environmental Sciences Center, 2630 Fanta Reed Road, La Crosse, WI 54603 USA; 6 Ecology & Evolutionary Biology Department, The University of Tennessee, 569 Dabney Hall, 1416 Circle Dr, Knoxville, TN 37996 USA; 7 Institute of Ecology, National Autonomous University of Mexico, University City, Coyoacán, 04510 Mexico City, CDMX Mexico; 8 Udall Center for Studies in Public Policy, The University of Arizona, 803 E 1St Street, Tucson, AZ 85719 USA

**Keywords:** Environmental impact, Conservation biology, Animal migration, Wind energy

## Abstract

Global expansion in wind energy development is a notable achievement of the international community’s effort to reduce carbon emissions during energy production. However, the increasing number of wind turbines have unintended consequences for migratory birds and bats. Wind turbine curtailment and other mitigation strategies can reduce fatalities, but improved spatial and temporal data are needed to identify the most effective way for wind energy development and volant migratory species to coexist. Mexican free-tailed bats (*Tadarida brasiliensis mexicana*) account for a large proportion of known bat fatalities at wind facilities in the southwestern US. We examined the geographic concordance between existing wind energy generation facilities, areas of high wind potential amenable for future deployment of wind facilities, and seasonally suitable habitat for these bats. We used ecological niche modeling to determine species distribution during each of 4 seasons. We used a multi-criteria GIS-based approach to produce a wind turbine siting suitability map. We identified seasonal locations with highest and lowest potential for the species’ probability of occurrence, providing a potential explanation for the higher observed fatalities during fall migration. Thirty percent of 33,606 wind turbines within the southwestern US occurred in highly suitable areas for Mexican free-tailed bats, primarily in west Texas. There is also broad spatial overlap between areas of high wind potential and areas of suitable habitat for Mexican free-tailed bats. Because of this high degree of overlap, our results indicate that post-construction strategies, such as curtailing the timing of operations and deterrents, would be more effective for bat conservation than strategic siting of new wind energy installations.

## Introduction

The international effort to reduce carbon emissions in response to climate change has contributed to a massive increase in wind energy development over the past 20 years, expanding global capacity for wind energy from 14 to 832 GW^1^. However, the production of energy from wind can also have detrimental effects on the environment^2–4^. The rotating blades and support towers of wind turbines placed along migratory routes have created a globally distributed hazard, potentially impacting birds and bats that rely on seasonal migrations^5–7^. To reduce animal fatalities, wind energy managers can practice curtailment, the temporary shutdown of wind turbines during low wind speeds, which may reduce bat fatalities by 40–93%^8^. Additional mitigation methods such as smart curtailment, which combines variables such as echolocation, with meteorological monitoring^9–11^, and bat deterrents^12^, may also be helpful. However, to meaningfully reduce conflicts between wind energy development and migratory species conservation, we need to know where and when wind turbines pose the highest risk to migratory species. In this paper, we use the example of a declining bat species^13–15^, the Mexican free-tailed bat (*Tadarida brasiliensis mexicana*), to demonstrate the potential for its coexistence alongside wind energy development, and to offer insight on the general challenge posed by wind energy to migratory species conservation.

Mexican free-tailed bats (MFTBs) range from southern Mexico to southern and western portions of the US^16^. Both male and female MFTBs overwinter in central and southern Mexico^17^, though new overwinter colonies have been found in central Texas^18^. By early spring, most females and some males depart to their summer breeding roosts in the southwestern US and northern Mexico^19^. They return to wintering grounds in Mexico beginning in early September^16^. Before most of the recent expansion of wind energy capacity, alterations to the landscape from intensive agriculture^20^, highly persistent organochlorine contaminants^21^, and the presence of DDT (dichloro-diphenyl-trichloroethane) in the environment^22^ is thought to have reduced the MFTB population from ~ 54 million in the 1950s to < 23 million by 2003^13,14^, though the population estimates were measured with considerable uncertainty. From the limited available data on impacts of wind turbines, it appears MFTBs may be disproportionately affected relative to other bat species. For instance, of 627 bat carcasses identified as belonging to eight bat species during 2,560 searches at 16 turbines in south Texas, 78% were MFTBs^12^. Furthermore, Weaver et. al ^23^ estimated 16 MFTB/MW/yr were killed at a wind facility near the US-Mexico border and showed MFTBs made up 76% of all bat carcasses found. Why MFTBs have high fatalities at wind facilities is not known, but MFTBs are widespread^24^, still locally numerous^25^, and fly long distances during migration and foraging, all of which may increase the probability of spatial conflict with wind turbines more than other species.

Distribution modeling can identify geographic areas where migratory birds and bats overlap with wind facilities^26,27^. The maximum entropy algorithm (Maxent) is often used to predict suitable habitat and can help to identify expected migratory corridors for bats^28^. This method can be used to identify how wind facilities may threaten bats. For example, Santos et al*.*^26^ combined distribution modeling and fatality data from wind facilities in Portugal to predict risk of fatality for four bat species (*Hypsugo savii, Nyctalus leisleri, Pipistrellus kuhlii,* and *Pipistrellus pipistrellus*). However, most studies of migratory species have to date not considered how habitat preferences might change across seasons, and as such may underestimate the potential distribution^7,26,29^, but see Starbuck et al.^27^ who modelled summer and fall bat distributions in northern Arizona. As a result of this omission, risk may often be underestimated. For migratory species, full-annual-cycle modeling that includes seasonal distributions is necessary to fully identify species distributions^30,31^.

Our objective in this paper is to assess the geographic concordance between existing wind facilities, areas of high wind potential amenable to future wind energy generation, and seasonally suitable habitat for MFTBs. This basic information allows us to identify areas where wind energy potential is high but MFTBs are unlikely to occur. Similarly, this research makes it possible to identify existing wind facilities located in areas of high suitability for MFTBs. Such facilities may be prime candidates for employing mitigation strategies. To our knowledge, this is the first paper to present spatially mapped data that could contribute to potential solutions for coexistence between expanding wind energy generation and transborder bat migrations.

## Methods

We used a multi-step approach to investigate the geographic concordance between MFTB distribution and wind turbine locations. First, we associated MFTB occurrence data to relevant environmental variables with the Maxent algorithm to estimate the species’ relative probability of presence during four seasons of its full annual cycle. Second, we gathered information on existing wind turbine locations in the US. We note that we could not find wind turbine locations in Mexico while conducting this research. Third, we analyzed the overlap between potential MFTB distribution and current wind turbine locations in the US. Fourth, we ranked wind facilities that might impact MFTBs using three levels of potential threat based on their relative probability of presence and the comprehensive wind turbine suitability map. Finally, we identified locations of high wind potential within areas of low to moderate suitability for MFTBs.

### Species occurrence data

We focused on seven US states (Texas, Oklahoma, Kansas, Colorado, New Mexico, Arizona, and California) and all states in Mexico within the MFTB’s range^16^. We downloaded MFTB occurrence data for 1970–2020 from the Global Biodiversity Information Facility (GBIF^32^) using those years that matched the WorldClim V2 historic data^33^ (see Environmental variables below). We only used preserved specimen data among the occurrences to avoid potential species misclassification error, resulting in 723 geographically unique MFTB records. Specimen records did not include the sex of individual bats.

To study the seasonality of habitat associations, we separated occurrence data into four seasons, specifically: 228 records in the spring (March–May), 236 records in the summer (June–August), 175 records in the fall (September–November), and 84 records in the winter (December-February). To lower the spatial autocorrelation in the model, we removed records with duplicate coordinates and randomly selected single occurrences from multiple occurrence records within 10 km via SDM toolbox v2.3^34^ in ArcMap10.5.1^35^. After reducing spatial autocorrelation, records by season were: 169 spring (March–May), 177 summer (June–August), 140 fall (September–November), and 54 winter (December-February).

### Environmental variables

The factors affecting MFTB seasonal distributions are not fully understood; we, therefore, selected environmental variables known to affect bat species distributions under similar conditions. Starbuck et al.^27^ used acoustic data to model MFTB distributions in northern Arizona. Because MFTBs were ubiquitous and detected at all sampling sites, their models lacked power in discriminating between areas of high likelihood of presence versus areas of absence, but indicated both temperature and precipation can affect MFTB distributions; other studies also showed bat occurrence is related to temperature and humidity^7,26,36,37^. Vapor pressure deficits (VPD) are associated with evaporative water loss in bats^37,38^. Solar radiation may also affect bat occurrences, possibly due to the formation of microclimates that persist nocturnally^39^. Land cover (e.g., urban) is a good predictor of migratory bat occurrence^40^. Lastly, low wind speed has also been found to be a good predictor of bat activity^41^.

For each season, we calculated mean temperature (ºC), mean solar radiation (KJm^-2^ day^-1^), mean water vapor pressure (kPa), mean wind speed (ms^-1^), and mean seasonal precipitation (mm) from 1970 to 2000 using Worldclim2^33^. We used mean values, not minimum and maximum to avoid multicollinearity^42,43^. We obtained land cover from the U.S. Geological Survey (USGS) North American Land Change Monitoring System (NALCMS^44^) as it includes both the US and Mexico. We assumed land cover did not change during the study period. The resolution of the NALCMS land cover data was 250 m, and the resolution for the remaining environmental variables was 30 s (Table S1). We aggregated land cover data to 1 km in R^45^. The final distribution modeling resolution was 1 km.

### Species distribution modeling

Based on the occurrences and environmental variables, we modeled MFTB distribution using the maximum entropy algorithm (Maxent V3.3.3k^46^) in R. We used Maxent because it is robust to small sample sizes and widely used for predicting species occurrence, including bats in unsurveyed areas^47,48^. Maxent uses machine learning to estimate the relative likelihood of a species’ presence by contrasting the distribution of relevant environmental variables at sample points (i.e., presence locations) to distributions at randomly selected background locations^46^. Maxent’s logistic output then provides estimates of probability of presence^49^, and locations can be ranked by habitat suitability based on training omission rate (percentage of training presences predicted as absences).

Prior to estimating models in Maxent, we examined environmental variables for multi-collinearity using Pearson correlations; all correlation coefficients were < 0.7 except the correlation between temperature and vapor pressure in winter (0.89). However, it has been noted collinearity is less of a problem for machine learning methods than it is for statistical methods^50^. The influence of sampling bias could also be lowered in Maxent through methods such as systematic sampling or spatial filtering^51,52^. To reduce the impact of sampling bias on Maxent models, we rarified the occurrence using a 10 km window.

Based on occurrence data, we set the feature class to linear, quadratic and product (LQP)^42,53^. We calculated the response curve to describe relative probabilities of presences across the range of values for the environmental variables^42,46^. We ran the ENMeval package to find the best regularization multiplier between 0 and 5 (at intervals of 0.5^54^). We chose the regularization multiplier with the lowest AIC score: spring:1, summer:1.5, fall: 1.5, and winter: 0.5. We used area under the receiver operating characteristics curve (AUC) values to evaluate model performance^55^.

To illustrate how relative probability of presences^42^ responded to each environmental predictor, we estimated additional models using environmental variables individually for each season following the same procedure but using default beta regularization multipliers. We used a 10% training omission rate to avoid potential effects caused by occasional observations or errors in the occurrence database^56^. We reclassified the suitability index based on the training omission error into three categories in real pixels: suitability index with < 10% training omission rate were considered “unsuitable habitat,” between 10 and 50% training omission rate were considered “low to moderate-suitability habitat,” and > 50% training omission rate were considered “high-suitability habitat”^57^.

### Wind turbine suitability map

We followed methods in Miller and Li^58^ to build a multi-criteria GIS wind-turbine siting suitability map (Table S2). Our criteria included: wind power class, slope, land cover, human population density, distance to transmission lines, and distance to major roads. The underlying wind power data excluded military land^59^, level 1 and 2 protected areas in USGS Protected Areas Database^60^, and areas within 1 km of airports^61^, identifying them as unsuitable areas for wind energy generation. All selected criteria were first converted into a raster data structure. We reclassified the data into ordinal suitability scores of 0–4, with 4 being the most suitable (Table S2). To be consistent with the projections of MFTB distribution modeling, we projected all layers into GRS 1980 Lambert conformal conic, and resampled all the raster datasets to a common 300-m cell size. We assigned a weight to each GIS layer to reflect the relative importance of each factor in the wind turbine siting suitability map. The weights for each factor were: wind power class (3), distance to roads (2), distance to transmission lines (2), land cover (2), slope (2), and human population density (1). We then summed the product of weighted suitability score for a final map.

We set wind turbine siting thresholds based on the distribution of the wind turbines across the suitability scores. The range of suitability scores was 0–48. Twenty percent of the wind turbines were located in areas with a suitability score < 32. Based on this threshold, we defined the areas with suitability scores 0–16 as “low suitability for siting” for wind turbines, scores 17–32 as “moderate suitability for siting,” and scores 33–48 as “high suitability for siting”^62^.

### Geographic overlap with existing wind facilities and wind facility suitability

We used the US Wind Turbine Database (version 2.2), comprising 33,606 locations of existing wind turbines in our study area^63^. This database contains visually verified coordinates of industrial-level wind turbines with capacity larger than 65 kW and blade size larger than 30 m^63^.

To map levels of overlap between modelled MFTB distribution and existing wind turbines, we used the “select by location” function in ArcMap 10.5.1 to map existing wind turbines located in the “unsuitable habitat”, “low to moderate-suitability habitat” and “high-suitability habitat” areas of the bat distribution maps generated in Maxent. We defined wind turbines located in unsuitable, low to moderate, and high-suitability habitat of bat distribution as having “unlikely”, “moderate”, and “high” potential impact, respectively, to the MFTB population.

We also examined geographic overlap between areas of “high siting suitability” for wind energy with the unsuitable, low to moderate, and high-suitability habitat within the MFTB distribution. We defined overlaps between high-suitability for siting wind facilities and high-suitability for MFTB habitat as “high potential-conflict areas”. We defined overlaps between high-suitability for siting wind facilities and low to moderate-suitability for MFTB habitat as “low potential-conflict areas”. We defined overlaps between high-suitability for wind siting and unsuitable areas for MFTB habitat as “minimum potential-conflict areas”. Lastly, we examined geographic overlap between existing wind turbines and areas of “high suitability for siting” wind energy and 100-km buffer of all major MFTB summer roosts in US^25^. The 100-km buffer represents a conservative estimate of how far MFTB fly from their roosts each night while foraging^64,65^.

## Results

### Mexican free-tailed bat distribution models

Our four seasonal distribution models indicated good overall predictive performance; spring testing AUC = 0.760 ± 0.029 (mean ± standard deviation), summer testing AUC = 0.623 ± 0.005, fall testing AUC = 0.610 ± 0.052 , and winter testing AUC = 0.798 ± 0.014.

**Figure 1:**
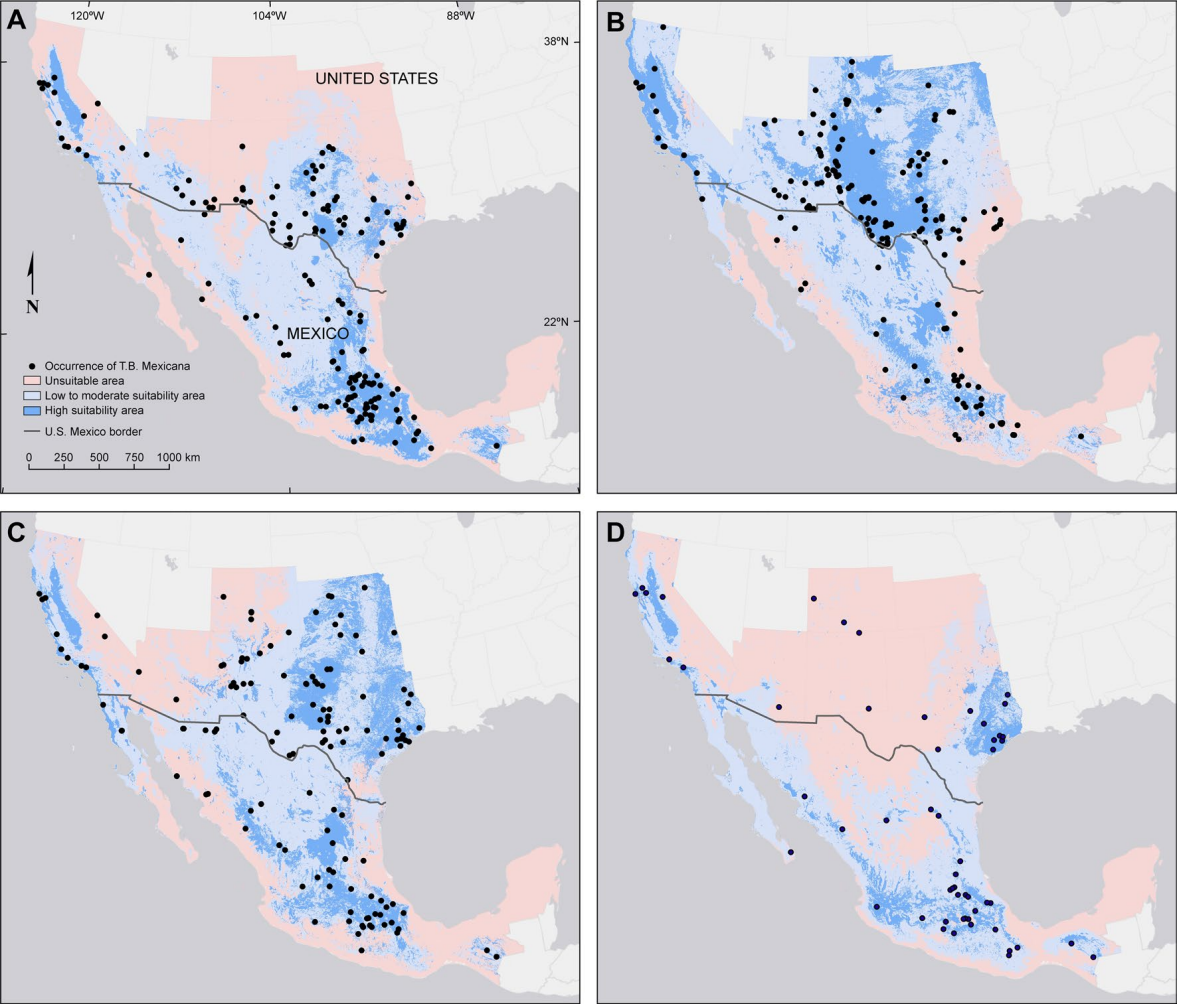
Maps of predicted suitable and unsuitable habitat areas for migratory Mexican free-tailed bats (*T. b. mexicana*) in California, Arizona, New Mexico, Colorado, Texas, Oklahoma, and Kansas, USA, and Mexico during spring (**A**), summer (**B**), fall (**C**), and winter (**D**). The distribution maps were modelled using Maxent and maps generated in ArcMap 10.6.1 (www.esri.com).

As expected from the north–south migration pattern of MFTBs, predicted locations of highly suitable areas of habitat differed by season (Fig. 1, Table S3). In the spring, when MFTBs migrate northward, the high-suitability areas were located in central and and some coastal locations of California, very few, small areas in central Arizona and New Mexico, and large areas scattered primarily across western and central Texas, and southern Mexico as well as a region of high suitability in northeastern Mexico connecting to southern areas in Texas. The summer model indicated suitable areas for MFTBs in central and coastal California, central and eastern Arizona, a the majority of central and eastern New Mexico, western to central Texas, and central Mexico*.* In the fall, when MFTBs migrate southward, the pattern was somewhat similar to the spring model, except that the fall model showed larger suitable areas in Texas, Oklahoma and Kansas. In winter, the model indicated central and coastal California, coastal Texas, and areas in central Mexico could be suitable areas for MFTBs though most bats have progressed south of the study area by this season.

### Importance of environmental variables for Mexican free-tailed bat distribution

The variables with the greatest contribution to MFTB distribution were land cover and water vapor pressure (Supplementary Fig. S1). Together, these two variables were responsible for an 84.8% contribution to the fall model, 84.1% contribution to the summer model, 51.0% contribution to the winter model, and 50.2% contribution to the spring model.

**Figure 2:**
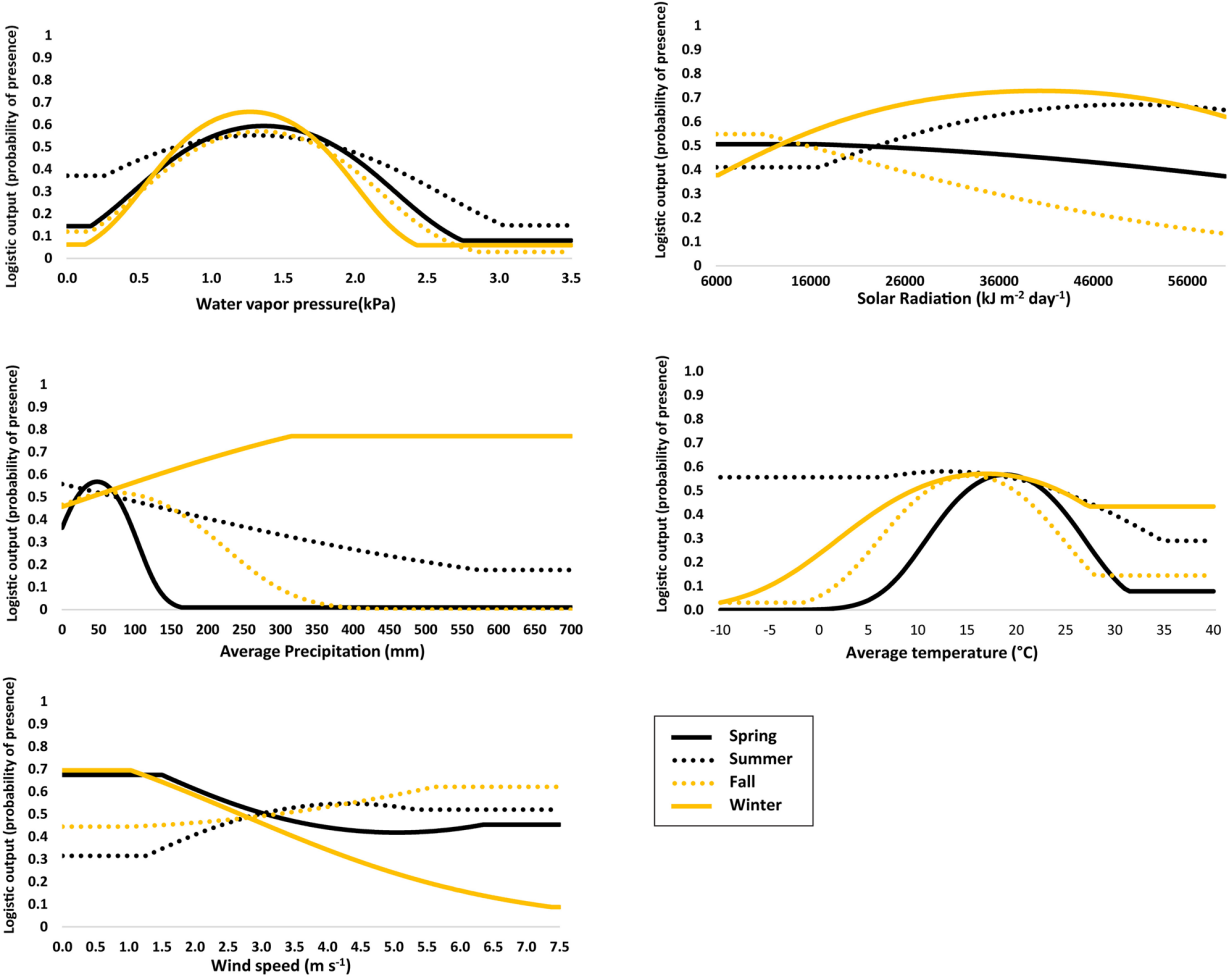
Seasonal response curves from Maxent analysis describing probabilities of occurrence for migratory Mexican free-tailed bats (*T. b. mexicana*) across a range of values for five environmental variables.

The response curve suggested urban areas were suitable habitat for MFTBs in all seasons (Fig. 2). In summer, temperate or sub-polar shrublands and tropical or sub-tropical grasslands were highly associated with suitability. High suitability occurred when water vapor pressure varied between 1.0 and 1.5 kPa. Most suitable average monthly temperatures were similar among seasons: spring (18 °C), summer (16 °C), fall (17 °C) and winter (17 °C). Monthly average precipitation of 50 and 70 mm in spring and fall, respectively, was associated with high suitability (Fig. 2).

### Wind turbine suitability map

**Figure 3:**
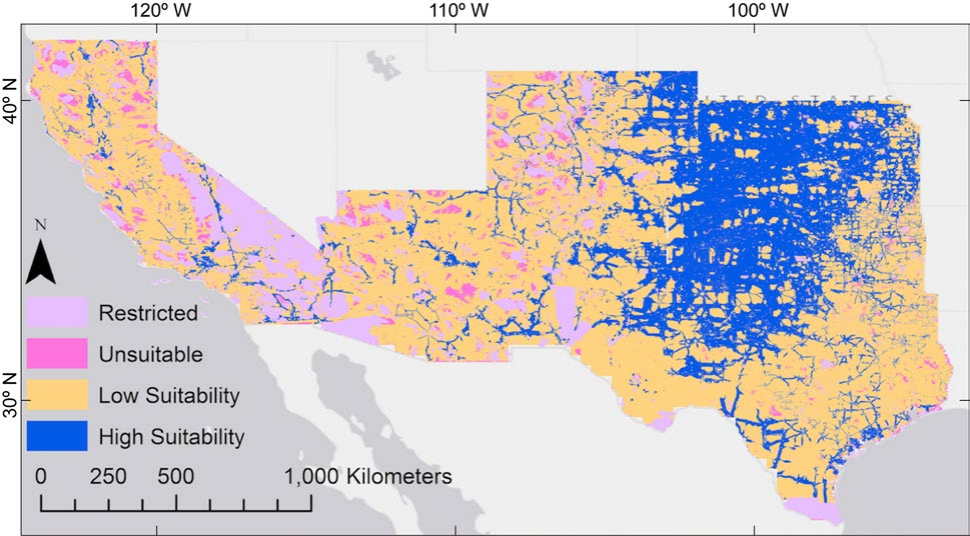
Suitability map for wind farm development in California, Arizona, New Mexico, Colorado, Texas, Oklahoma, and Kansas, USA (The weights for each factor were: wind power class (3), distance to roads (2), distance to transmission lines (2), land cover (2), slope (2), and population density (1)). The GIS model and map was generated in in ArcMap 10.6.1 (www.esri.com).

We predicted major areas of high suitability for siting wind turbines in Kansas, Oklahoma, northwestern and coastal Texas, eastern New Mexico and eastern Colorado (Fig. 3). Our prediction showed broad agreement with wind potential maps developed by NREL^66,67^. The major factors dominating suitability for wind turbines were wind power class and distance to transmission lines.

### Geographic overlap

In summer and fall, the proportion of wind turbines located in the suitable areas for MFTB were relatively high compared to spring and winter (Table 1). Approximately one-third (36.3%) of the 33,606 wind turbines within the study area, most in western Texas, western Oklahoma and Kansas, occurred in highly suitable summer areas; 29.5% of the total turbines were located in highly suitable fall areas (mostly northwestern Texas, western Oklahoma and Kansas); 10.8% of the total turbines were located in highly suitable spring areas (mostly western Texas and California); and only 0.3% of the total wind turbines were located in highly suitable winter areas (mostly coastal California and southern Texas; Table 1).

**Table 1:** Number of existing wind turbines located in the suitable areas for *T.b. mexicana* in the southwestern US.

	Unsuitable habitat (Percentage of total wind turbines)	Low to moderate suitability habitat (Percentage of total wind turbines)	High suitability habitat (Percentage of total wind turbines)
Spring	12,224 (36.4%)	17,756 (52.8%)	3,626 (10.8%)
Summer	1,820 (5.4%)	19,580 (58.3%)	12,200 (36.3%)
Fall	8,471 (25.2%)	15,219 (45.3%)	9,913 (29.5%)
Winter	27,616 (82.2%)	5,894 (17.5%)	96 (0.3%)

**Figure 4:**
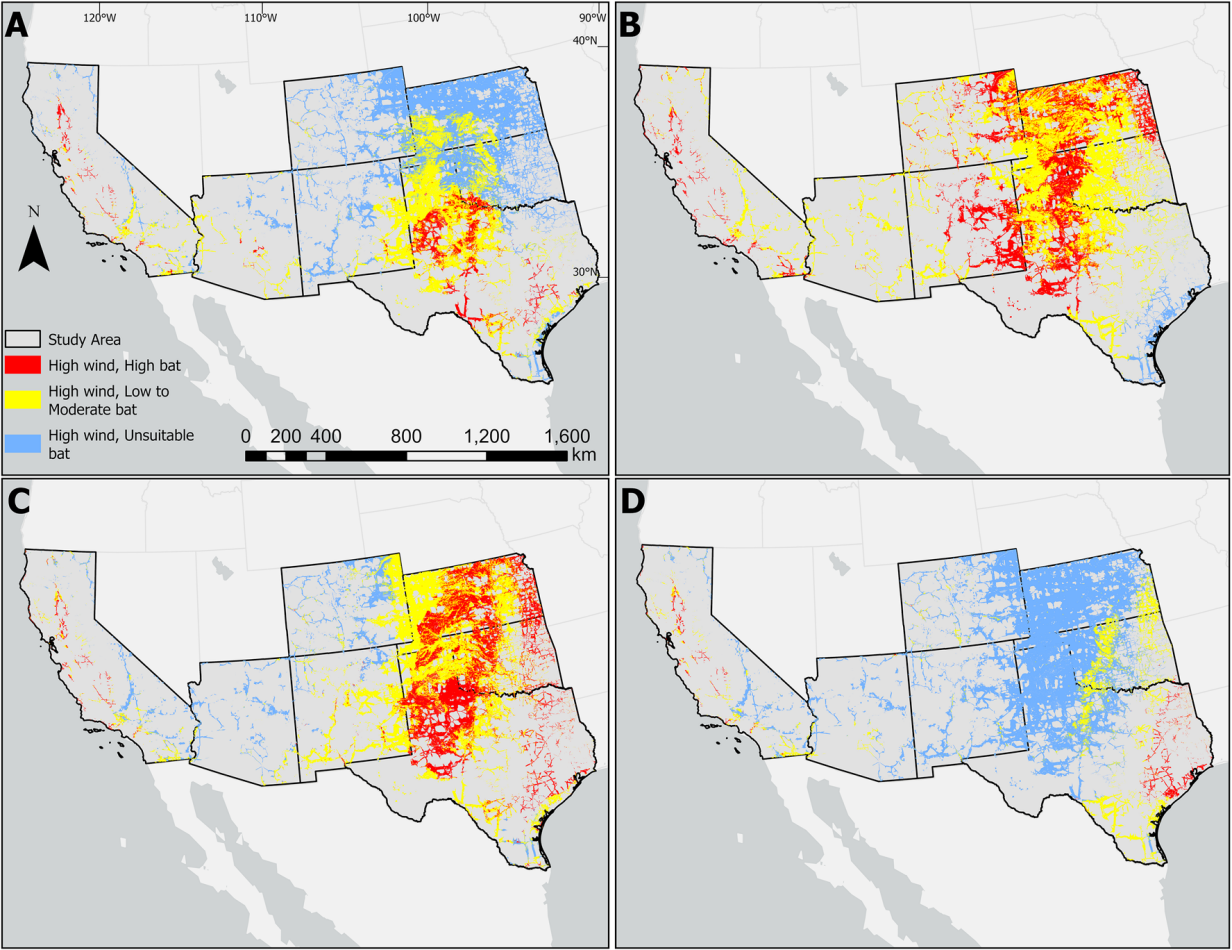
Maps showing predicted areas where highly suitable sites for wind turbines overlap with three levels of Mexican free-tailed bat (*T. b. mexicana*) habitat suitability in California, Arizona, New Mexico, Colorado, Texas, Oklahoma, and Kansas, USA during spring (**A**), summer (**B**), fall (**C**), and winter (**D**). The overlay analyses and maps were generated in ArcMap 10.6.1 (www.esri.com).

Northwestern Texas was the area with highest potential conflict with MFTBs in the spring, summer, and fall (Fig. 4). Eastern Oklahoma and eastern Kansas were the areas with least potential conflict with MFTBs in spring and winter (Fig. 4). There were few locations with consistently occurring high wind potential and low bat habitat suitability across all seasons, indicating low potential conflict areas were rare. While many locations met these criteria in the winter (Fig. 4d), in the summer bats were widely distributed across nearly all highly suitable wind turbine locations (Fig. 4b).

## Discussion

To our knowledge, this is the first study to identify the full annual cycle distribution of MFTB habitat across a large portion of its range in the US. We used these distributions to examine the spatial congruence between MFTB and current and potential wind energy because fatality data suggest MFTBs are killed at relatively higher numbers than other species. Recent studies have employed seasonal-distribution modeling for other migratory species. For example, Hayes et al.^68^ also used Maxent to produce a seasonal distribution model of hoary bats compared to the US wind turbine database and Starbuck et al.^27^ compared fall and summer bat distributions to wind power classes in northeast Arizona. However, neither study formally examined changes in the potential risk and the seasonal distribution of bat fatality data. Here, we further compared the response curve to show MFTB ecological niches across seasons. Other recent studies have also recommended considering phenology data in distribution models^7,69^.

### Comparisons with current fatality data

Bat fatalities generally increase during August and September both nationally and across different regions of the US^70,71^. Lloyd et al.^70^ found peaks in MFTB carcasses in September to October in two, level 2 ecoregions overlapping our study extent. Our study area included the Pacific Southwest and Southwest regions reported in^71^ and in these regions, all bat fatalities were elevated from August to October with a peak in September. The majority of observed counts of fatalities in these two regions were attributed to MFTBs^71^.

Many bats, including MFTBs migrate during the fall and a number of hypotheses attempt to explain the increase in fatalities during this season^72,73^. Our results suggest a variant of Cryan and Barclay’s (2009) “coincidental cause” may explain seasonal patterns of MFTB fatalities in the southwestern US; during migration, shifts in MFTB distribution result in more individuals interacting with more turbines. Another possible reason could be that fall migrations involve larger populations as they include young of the year, and those young individuals lack knowledge of the landscape (including wind facilities). Note that the patterns we observed are not exclusive of other processes, such as bat attraction to turbines^72,73^. Fatality studies that use isotopes or genetics to understand the origin of killed animals^74,75^ in addition to understanding spatio-temporal patterns in the age structure^76^ and sex ratios^77,78^ of fatalities could add to our understanding. For example, LiCari et al.^78^ (Fig. 4) suggest female skewed sex ratios of carcasses at the two south Texas wind facilities during the fall migration, though they did not anlayze the data by month or season.

Our results show that habitat in Texas plays a vital role in the spring and fall, when the bats are migrating and as a result are likely at greatest risk during this season; this result is consistent with previous studies indicating that MFTBs may account for up to 76% of the total bat fatalities in the southwestern US^12,71,79^. Our finding is consistent with previous MFTB studies, which reported more fatalities in the fall migration than the spring migration^71,72^. Our results indicate that 9,913 wind turbines were located in areas that are highly suitable for MFTBs during the fall migratory season, providing a potential explanation for why fall migration has much higher fatalities than spring migration (when less than a third as many wind turbines co-occurred).

The density of wind turbines overlapping with areas of high suitability for MFTBs was an order of magnitude higher in the fall and summer than winter and spring, coincident with patterns of monthly fatality data^71^ given our definition of seasons, where both summer and fall include months of high fatality. Hayes et al.^68^ reported that potential suitable habitat for hoary bats (*Lasiurus cinereus*) migrating in fall was more dispersed than in other seasons, the mating behavior and foraging at wind turbines possibly explaining higher hoary bat fatalities in fall compared with spring and other seasons. One way to further test this hypothesis is to examine spatio-temporal patterns in bat fatalities. It may be possible to determine patterns in both the wind farm-level fatality rate and the distribution of wind facilities having fatalities. The increase in fall fatalities may be caused by fatalities occurring across a larger number, and/or broader spatial distribution of wind facilities or because fatalities increase at individual wind facilities. The two patterns are not mutually exclusive, but understanding how they covary through time will help determine causes of fatalities in bats.

### Limitations

Future researchers could consider comparing the results of species distribution models with ground validation^48,80^ and occupancy models^27^. We used thresholds to reclassify the suitability index from Maxent for MFTBs because resources for conservation tend to be limited and decisions about the use of curtailment and deterrents must take high opportunity costs into account. We identified the areas with high probability and suitability for MFTB habitat and migration while taking these real-world limitations into account.

### Management implications

Siting wind turbines in areas of low species habitat suitability or away from features that increase occupancy has been suggested as one method to reduce fatality risk for bats^27,81^. However, our results indicate that there are very few areas in the southwestern US suited for wind energy and unsuitable for MFTB across all seasons (Fig. 4). As such, post-construction strategies such as curtailment and deterrents systems may be more realistic for MFTB conservation than strategies focused on siting new wind turbines away from bats. Wind turbine curtailment is likely one of most effective measures for mitigating bat fatalities from wind turbines^82^, and tests of ultrasonic acoustic deterrents have shown promise^12^. It may also be possible to use seasonal maps like ours (Fig. 4) to site facilities so they only require curtailment during certain seasons.

It would be valuable to estimate the cumulative impact of curtailment on MFTBs in conjunction with its impacts on energy production, costs of electricity, and the ability to meet net-zero energy production goals. Curtailment can reduce bat fatalities in other bat species^82^, yet doing so may raise the costs of electricity and require more installed turbines to meet greenhouse gas reduction targets. Our models show high conflict between suitable areas for MFTBs and wind facilities in the summer and fall migrating seasons, but much lower overlap in other seasons, which suggests seasonal curtailment might be a key strategy to reduce fatalities while minimizing loss of energy production. Further study is needed to assess the relationship between wind speed and MFTB fatalities to determine the optimal wind cut-in speed (the lowest wind speed for wind turbines to generate power) and other environmental conditions correlated with bat foraging and fatality at wind turbines^9,12^.

The recently passed Inflation Reduction Act of 2021 and the Infrastructure Investment and Jobs Act of 2021 (referred to as the Bipartisan Infrastructure Law) will generate > $430 billion in tax credits, and other incentives for decarbonization and modernization of the US energy system^83^. These investments will spur new installations. In addition, energy production tax credits and technology allowing high performance in low wind conditions^84^ may result in turbines being sited in locations currently considered economically unviable. Owners/operators of new wind facilities will obviously aim to maximize return on investment, so optimal locations will still be preferred, but the new economic and technological horizons for wind suggest more, not less overlap with MFTBs, further arguing for advanced curtailment approaches and deterrents.

## Conclusion

Wind energy development has seen rapid worldwide growth, yet gaps exist in our understanding of how wind energy affects bats^85^. Our study provides an approach for understanding how seasonal population dynamics may change how and where species interact with existing wind turbines. We provide a possible answer to the question of why more fatalities occur for MFTBs during fall migration than spring migration^72^ and suggest approaches to further test hypotheses about fatalities during migration. The spatial patterns of overlap between MFTBs and current/potential wind turbines we identified could help reduce MFTB fatalities by informing bat fatality monitoring programs and curtailment practices. Our models also show potential conflict between suitable areas for MFTBs and wind facilities in the fall migration season, which suggests a siting strategy for new turbines is unlikely to reduce fatalities. Our data suggest post-construction strategies such as curtailment and deterrent systems are more likely to reduce fatalities because fall and summer distributions overlap existing turbines and areas highly suitable for more wind energy.

### Supplementary Information

Supplementary Information.

## Data Availability

The datasets analysed during the current study are all publicly available. See Table S1 for information and persistent web links to each dataset.

## References

[1] International Energy Agency. *World Energy Outlook, 2022*. https://www.iea.org/reports/world-energy-outlook-2022 (2022).

[2] Schuster, E., Bulling, L. & Köppel, J. Consolidating the state of knowledge: a synoptical review of wind energy’s wildlife effects. *Environ. Manag.* **56**, 300–331 (2015). 10.1007/s00267-015-0501-5 PMC4493795 25910869

[3] Popescu, V. D. *et al.* Quantifying biodiversity trade-offs in the face of widespread renewable and unconventional energy development. *Sci. Rep.* **10**, 7603 (2020). 32371910 10.1038/s41598-020-64501-7 PMC7200705

[4] Wang, S., Wang, S. & Smith, P. Ecological impacts of wind farms on birds: questions, hypotheses, and research needs. *Renew. Sustain. Energy Rev.* **44**, 599–607 (2015). 10.1016/j.rser.2015.01.031

[5] Katzner, T. E. *et al.* Golden eagle fatalities and the continental-scale consequences of local wind-energy generation. *Conserv. Biol.* **31**, 406–415 (2017). 27677518 10.1111/cobi.12836

[6] Frick, W. F. *et al.* Fatalities at wind turbines may threaten population viability of a migratory bat. *Biol. Conserv.* **209**, 172–177 (2017). 10.1016/j.biocon.2017.02.023

[7] Smeraldo, S. *et al.* Modelling risks posed by wind turbines and power lines to soaring birds: the black stork (*Ciconia nigra*) in Italy as a case study. *Biodivers. Conserv.* **29**, 1959–1976 (2020). 10.1007/s10531-020-01961-3

[8] Arnett, E. B., Huso, M. M., Schirmacher, M. R. & Hayes, J. P. Altering turbine speed reduces bat mortality at wind-energy facilities. *Front. Ecol. Environ.* **9**, 209–214 (2011). 10.1890/100103

[9] Hayes, M. A. *et al.* A smart curtailment approach for reducing bat fatalities and curtailment time at wind energy facilities. *Ecol. Appl.* **29**, e01881 (2019). 30939226 10.1002/eap.1881

[10] Martin, C. M., Arnett, E. B., Stevens, R. D. & Wallace, M. C. Reducing bat fatalities at wind facilities while improving the economic efficiency of operational mitigation. *J. Mammal.* **98**, 378–385 (2017). 10.1093/jmammal/gyx005

[11] Weller, T. J. & Baldwin, J. A. Using echolocation monitoring to model bat occupancy and inform mitigations at wind energy facilities. *J. Wildl. Manag.* **76**, 619–631 (2012). 10.1002/jwmg.260

[12] Weaver, S. P., Hein, C. D., Simpson, T. R., Evans, J. W. & Castro-Arellano, I. Ultrasonic acoustic deterrents significantly reduce bat fatalities at wind turbines. *Global Ecol. Conserv.*10.1016/j.gecco.2020.e01099 (2020). 10.1016/j.gecco.2020.e01099

[13] McCracken, G. F. Estimates of population sizes in summer colonies of Brazilian free-tailed bats (*Tadarida brasiliensis*). in *Monitoring trends in bat populations of the United States and territories: problems and prospects* (eds. O’Shea, T. J. & Bogan, M. A.) 21–30 (United States Geological Survey, Biological Resources Discipline, Sciences Division, Information and Technology Report USGS/BRD/ITR-2003- 003, Reston, Virginia., 2003).

[14] Betke, M. *et al.* Thermal imaging reveals significantly smaller Brazilian free-tailed bat colonies than previously estimated. *J. Mammal.* **89**, 18–24 (2008). 10.1644/07-MAMM-A-011.1

[15] Betke, M. Erratum. *J. Mammal.* **90**, 783 (2009). 10.1644/1545-1542-90.3.783b

[16] Russell, A. L., Medellín, R. A. & Mccracken, G. F. Genetic variation and migration in the Mexican free-tailed bat (*Tadarida brasiliensis mexicana*). *Mol. Ecol.* **14**, 2207–2222 (2005). 15910338 10.1111/j.1365-294X.2005.02552.x

[17] Bernardo, V. R. & Cockrum, E. L. Migration in the guano bat *Tadarida brasiliensis mexicana* (Saussure). *J. Mammal.* **43**, 43–64 (1962). 10.2307/1376879

[18] Weaver, S. P., Simpson, T. R., Baccus, J. T. & Weckerly, F. W. Baseline population estimates and microclimate data for newly established overwintering Brazilian free-tailed bat colonies in central Texas. *swna* **60**, 151–157 (2015). 10.1894/SWNAT-D-14-00022.1

[19] Federico, P. *et al.* Brazilian free-tailed bats as insect pest regulators in transgenic and conventional cotton crops. *Ecol. Appl.* **18**, 826–837 (2008). 18536245 10.1890/07-0556.1

[20] Davidai, N., Westbrook, J. K., Lessard, J.-P., Hallam, T. G. & McCracken, G. F. The importance of natural habitats to Brazilian free-tailed bats in intensive agricultural landscapes in the Winter Garden region of Texas, United States. *Biol. Conserv.* **190**, 107–114 (2015). 10.1016/j.biocon.2015.05.015

[21] Geluso, K. N., Altenbach, J. S. & Wilson, D. E. Organochlorine residues in young mexican free-tailed bats from several roosts. *Am. Midl. Nat.* **105**, 249–257 (1981). 10.2307/2424743

[22] Clark, D. R. Jr. DDT and the decline of free-tailed bats (*Tadarida brasiliensis*) at Carlsbad Cavern, New Mexico. *Arch. Environ. Contam. Toxicol.* **40**, 537–543 (2001). 11525497 10.1007/s002440010207

[23] Weaver, S. P., Jones, A. K., Hein, C. D. & Castro-Arellano, I. Estimating bat fatality at a Texas wind energy facility: implications transcending the United States-Mexico border. *J. Mammal.* **101**, 1533–1541 (2020). 10.1093/jmammal/gyaa132

[24] Wilkins, K. T. Tadarida brasiliensis. *Mamm. Species*10.2307/3504148 (1989). 10.2307/3504148

[25] Wiederholt, R. *et al.* Moving across the border: modeling migratory bat populations. *Ecosphere* **4**, art114 (2013). 10.1890/ES13-00023.1

[26] Santos, H., Rodrigues, L., Jones, G. & Rebelo, H. Using species distribution modelling to predict bat fatality risk at wind farms. *Biol. Conserv.* **157**, 178–186 (2013). 10.1016/j.biocon.2012.06.017

[27] Starbuck, C. A., Dickson, B. G. & Chambers, C. L. Informing wind energy development: land cover and topography predict occupancy for Arizona bats. *Plos One* **17**, e0268573 (2022). 35657796 10.1371/journal.pone.0268573 PMC9165840

[28] Bond, M. L., Bradley, C. M., Kiffner, C., Morrison, T. A. & Lee, D. E. A multi-method approach to delineate and validate migratory corridors. *Landscape Ecol.* **32**, 1705–1721 (2017). 10.1007/s10980-017-0537-4

[29] Feng, X., Castro, M. C., McBee, K. & Papeş, M. Hiding in a cool climatic niche in the tropics? An assessment of the ecological biogeography of hairy long-nosed armadillos (*Dasypus pilosus*). *Trop. Conserv. Sci.* **10**, 1940082917697249 (2017). 10.1177/1940082917697249

[30] Hostetler, J. A., Sillett, T. S. & Marra, P. P. Full-annual-cycle population models for migratory birds. *Auk* **132**, 433–449 (2015). 10.1642/AUK-14-211.1

[31] Erickson, R. A., Thogmartin, W. E., Diffendorfer, J. E., Russell, R. E. & Szymanski, J. A. Effects of wind energy generation and white-nose syndrome on the viability of the Indiana bat. *PeerJ* **4**, e2830 (2016). 28028486 10.7717/peerj.2830 PMC5183089

[32] GBIF Home Page. https://www.gbif.org/ (2021).

[33] Fick, S. E. & Hijmans, R. J. WorldClim 2: new 1-km spatial resolution climate surfaces for global land areas. *Int. J. Climatol.* **37**, 4302–4315 (2017). 10.1002/joc.5086

[34] Brown, J. L., Bennett, J. R. & French, C. M. SDMtoolbox 2.0: the next generation Python-based GIS toolkit for landscape genetic, biogeographic and species distribution model analyses. *PeerJ* **5**, e4095 (2017). 29230356 10.7717/peerj.4095 PMC5721907

[35] Esri. ArcMap: Release 10.5.1. Environmental systems research institute (2018).

[36] Adams, R. A. & Hayes, M. A. Water availability and successful lactation by bats as related to climate change in arid regions of western North America. *J. Anim. Ecol.* **77**, 1115–1121 (2008). 18684132 10.1111/j.1365-2656.2008.01447.x

[37] Rebelo, H., Tarroso, P. & Jones, G. Predicted impact of climate change on European bats in relation to their biogeographic patterns. *Global Change Biol.* **16**, 561–576 (2010). 10.1111/j.1365-2486.2009.02021.x

[38] Webb, P. I., Speakman, J. R. & Racey, P. A. Evaporative water loss in two sympatric species of vespertilionid bat, *Plecotus auritus* and *Myotis daubentoni*: relation to foraging mode and implications for roost site selection. *J. Zool.* **235**, 269–278 (1995). 10.1111/j.1469-7998.1995.tb05143.x

[39] De La Cruz, J. L. & Ward, R. L. Summer-habitat suitability modeling of *Myotis sodalis* (Indiana Bat) in the eastern mountains of West Virginia. *Northeast. Nat.* **23**, 100–117 (2016). 10.1656/045.023.0107

[40] Lundy, M., Montgomery, I. & Russ, J. Climate change-linked range expansion of Nathusius’ pipistrelle bat, *Pipistrellus nathusii* (Keyserling & Blasius, 1839). *J. Biogeogr.* **37**, 2232–2242 (2010). 10.1111/j.1365-2699.2010.02384.x

[41] Cryan, P. M. & Brown, A. C. Migration of bats past a remote island offers clues toward the problem of bat fatalities at wind turbines. *Biol. Conserv.* **139**, 1–11 (2007). 10.1016/j.biocon.2007.05.019

[42] Merow, C., Smith, M. J. & Silander, J. A. Jr. A practical guide to MaxEnt for modeling species’ distributions: what it does, and why inputs and settings matter. *Ecography* **36**, 1058–1069 (2013). 10.1111/j.1600-0587.2013.07872.x

[43] Petitpierre, B., Broennimann, O., Kueffer, C., Daehler, C. & Guisan, A. Selecting predictors to maximize the transferability of species distribution models: lessons from cross-continental plant invasions. *Global Ecol. Biogeogr.* **26**, 275–287 (2017). 10.1111/geb.12530

[44] CEC (Commission for Environmental Cooperation). 2010 Land Cover of North America at 250 meters, version 2. (2013).

[45] R Core Team. R: A language and environment for statistical computing. R Foundation for Statistical Computing, Vienna, Austria. URL https://www.R-project.org/.statistical computing, Vienna, Austria. ISBN 3-900051-07-0. (2018).

[46] Phillips, S. J., Anderson, R. P. & Schapire, R. E. Maximum entropy modeling of species geographic distributions. *Ecol. Modell.* **190**, 231–259 (2006). 10.1016/j.ecolmodel.2005.03.026

[47] Roscioni, F. *et al.* A modelling approach to infer the effects of wind farms on landscape connectivity for bats. *Landsc. Ecol.* **29**, 891–903 (2014). 10.1007/s10980-014-0030-2

[48] Rebelo, H. & Jones, G. Ground validation of presence-only modelling with rare species: a case study on barbastelles *Barbastella barbastellus* (Chiroptera: Vespertilionidae). *J. Appl. Ecol.* **47**, 410–420 (2010). 10.1111/j.1365-2664.2009.01765.x

[49] Phillips, S. J. & Dudík, M. Modeling of species distributions with Maxent: new extensions and a comprehensive evaluation. *Ecography* **31**, 161–175 (2008). 10.1111/j.0906-7590.2008.5203.x

[50] Elith, J. *et al.* A statistical explanation of MaxEnt for ecologists. *Divers. Distrib.* **17**, 43–57 (2011). 10.1111/j.1472-4642.2010.00725.x

[51] Kramer-Schadt, S. *et al.* The importance of correcting for sampling bias in MaxEnt species distribution models. *Divers. Distrib.* **19**, 1366–1379 (2013). 10.1111/ddi.12096

[52] Fourcade, Y., Engler, J. O., Rödder, D. & Secondi, J. Mapping species distributions with MAXENT using a geographically biased sample of presence data: a performance assessment of methods for correcting sampling bias. *Plos One* **9**, e97122 (2014). 24818607 10.1371/journal.pone.0097122 PMC4018261

[53] Papeş, M., Cuzin, F. & Gaubert, P. Niche dynamics in the European ranges of two African carnivores reflect their dispersal and demographic histories. *Biol. J. Linnean Soc.* **114**, 737–751 (2015). 10.1111/bij.12477

[54] Muscarella, R. *et al.* ENMeval: an R package for conducting spatially independent evaluations and estimating optimal model complexity for Maxent ecological niche models. *Methods Ecol. Evolut.* **5**, 1198–1205 (2014). 10.1111/2041-210X.12261

[55] Fielding, A. H. & Bell, J. F. A review of methods for the assessment of prediction errors in conservation presence/absence models. *Environ. Conserv.* **24**, 38–49 (1997). 10.1017/S0376892997000088

[56] Peterson, A. T. *et al.**Ecological Niches and Geographic Distributions (MPB-49)* (Princeton University Press, Princeton, 2011).

[57] Liu, C., White, M. & Newell, G. Selecting thresholds for the prediction of species occurrence with presence-only data. *J. Biogeogr.* **40**, 778–789 (2013). 10.1111/jbi.12058

[58] Miller, A. & Li, R. A geospatial approach for prioritizing wind farm development in northeast Nebraska, USA. *ISPRS Int. J. Geo-Inf.* **3**, 968–979 (2014). 10.3390/ijgi3030968

[59] DOD (US Department of Defense). *Military Installations, Ranges, and Training Areas*. https://www.globalsecurity.org/military/library/report/2009/090930_fy10_baseline_dod_bsr.pdf (2010).

[60] USGS (US Geological Survey). *Gap Analysis Project (GAP), 2018, Protected Areas Database of the United States (PAD-US)*. 10.5066/P955KPLE (2018).

[61] NREL (National Renewable Energy Lab). *Wind Prospector*. https://maps.nrel.gov/wind-prospector/ (2018).

[62] Değirmenci, S., Bingöl, F. & Sofuoglu, S. C. MCDM analysis of wind energy in Turkey: decision making based on environmental impact. *Environ. Sci. Pollut. Res.* **25**, 19753–19766 (2018). 10.1007/s11356-018-2004-4 29736652

[63] Rand, J. T. *et al.* A continuously updated, geospatially rectified database of utility-scale wind turbines in the United States. *Sci. Data* **7**, 1–12 (2020). 31932591 10.1038/s41597-020-0353-6 PMC6957502

[64] Horn, J. W. & Kunz, T. H. Analyzing NEXRAD doppler radar images to assess nightly dispersal patterns and population trends in Brazilian free-tailed bats (*Tadarida brasiliensis*). *Integr. Comp. Biol.* **48**, 24–39 (2008). 21669770 10.1093/icb/icn051

[65] McCracken, G. F. *et al.* Airplane tracking documents the fastest flight speeds recorded for bats. *R. Soc. Open Sci.* **3**, 160398 (2016). 28018618 10.1098/rsos.160398 PMC5180116

[66] Lopez, A. *et al.* Land use and turbine technology influences on wind potential in the United States. *Energy* **223**, 120044 (2021). 10.1016/j.energy.2021.120044

[67] Mai, T., Lopez, A., Mowers, M. & Lantz, E. Interactions of wind energy project siting, wind resource potential, and the evolution of the U.S. power system. *Energy* **223**, 119998 (2021). 10.1016/j.energy.2021.119998

[68] Hayes, M. A., Cryan, P. M. & Wunder, M. B. Seasonally-dynamic presence-only species distribution models for a cryptic migratory bat impacted by wind energy development. *Plos One* **10**, e0132599 (2015). 26208098 10.1371/journal.pone.0132599 PMC4514827

[69] Williams, H. M., Willemoes, M. & Thorup, K. A temporally explicit species distribution model for a long distance avian migrant, the common cuckoo. *J. Avian Biol.* **48**, 1624–1636 (2017). 10.1111/jav.01476

[70] Lloyd, J. D., Butryn, R., Pearman-Gillman, S. & Allison, T. D. Seasonal patterns of bird and bat collision fatalities at wind turbines. *Plos One* **18**, e0284778 (2023). 37163474 10.1371/journal.pone.0284778 PMC10171668

[71] AWWI (American Wind Wildlife Institute. *A Summary of Bat Fatality Data in a Nationwide Database*. https://rewi.org/wp-content/uploads/2019/02/AWWI-Bat-Technical-Report_07_25_18_FINAL.pdf (2008).

[72] Cryan, P. M. & Barclay, R. M. R. Causes of bat fatalities at wind turbines: hypotheses and predictions. *J. Mammal.* **90**, 1330–1340 (2009). 10.1644/09-MAMM-S-076R1.1

[73] Guest, E. E. *et al.* An updated review of hypotheses regarding bat attraction to wind turbines. *Animals* **12**, 343 (2022). 35158666 10.3390/ani12030343 PMC8833423

[74] Cryan, P. M., Stricker, C. A. & Wunder, M. B. Continental-scale, seasonal movements of a heterothermic migratory tree bat. *Ecol. Appl.* **24**, 602–616 (2014). 24988763 10.1890/13-0752.1

[75] Vander Zanden, H. B. *et al.* The geographic extent of bird populations affected by renewable-energy development. *Conserv. Biol.* **38**, e14191 (2024). 38180844 10.1111/cobi.14191

[76] Wilkinson, G. S. *et al.* DNA methylation predicts age and provides insight into exceptional longevity of bats. *Nat. Commun.* **12**, 1615 (2021). 33712580 10.1038/s41467-021-21900-2 PMC7955057

[77] Korstian, J. M., Hale, A. M., Bennett, V. J. & Williams, D. A. Advances in sex determination in bats and its utility in wind-wildlife studies. *Mol. Ecol. Resour.* **13**, 776–780 (2013). 23647806 10.1111/1755-0998.12118

[78] LiCari, S. T. *et al.* Understanding fatality patterns and sex ratios of Brazilian free-tailed bats (*Tadarida brasiliensis*) at wind energy facilities in western California and Texas. *PeerJ* **11**, e16580 (2023). 38084143 10.7717/peerj.16580 PMC10710772

[79] Arnett, E. B. *et al.* Patterns of bat fatalities at wind energy facilities in North America. *J. Wildl. Manag.* **72**, 61–78 (2008). 10.2193/2007-221

[80] Fois, M., Fenu, G., Cuena Lombraña, A., Cogoni, D. & Bacchetta, G. A practical method to speed up the discovery of unknown populations using species distribution models. *J. Nat. Conserv.* **24**, 42–48 (2015). 10.1016/j.jnc.2015.02.001

[81] Peste, F. *et al.* How to mitigate impacts of wind farms on bats? A review of potential conservation measures in the European context. *Environ. Impact Assess. Rev.* **51**, 10–22 (2015). 10.1016/j.eiar.2014.11.001

[82] Adams, E. M., Gulka, J. & Williams, K. A. A review of the effectiveness of operational curtailment for reducing bat fatalities at terrestrial wind farms in North America. *Plos One* **16**, e0256382 (2021). 34788295 10.1371/journal.pone.0256382 PMC8598023

[83] Steinberg, D. C. *et al. Evaluating Impacts of the Inflation Reduction Act and Bipartisan Infrastructure Law on the U.S. Power System*. https://www.osti.gov/biblio/1962552 (2023) 10.2172/1962552.

[84] Roberts, O., Williams, T., Lopez, A., Maclaurin, G. & Eberle, A. *Exploring the Impact of Near-Term Innovations on the Technical Potential of Land-Based Wind Energy*. https://www.osti.gov/biblio/1963405 (2023) 10.2172/1963405.

[85] Allison, T. D. *et al.* Impacts to wildlife of wind energy siting and operation in the United States. *Issues Ecol.* **23**, 2–18 (2019).

