# Supplementary material for: Potential for spatial coexistence of a transboundary migratory species and wind energy development
Source: Sci Rep. 2024 Jul 24;14:17050. doi: 10.1038/s41598-024-66490-3 (PMC11269593; doi:10.1038/s41598-024-66490-3)
Supplement: Supplementary file 1 — Supplementary Information. [file 41598_2024_66490_MOESM1_ESM.docx]

**Supporting Materials for:**

**Potential for spatial coexistence of a transboundary migratory species and wind energy development**

Ta-Ken Huang^1^*^,^*^2^, Xiao Feng^3^, Jonathan J. Derbridge^2^, Kaitlin Libby^2^, Jay E. Diffendorfer*^4^, Wayne E. Thogmartin^5^, Gary McCracken^6^, Rodrigo Medellin^7^, and Laura López-Hoffman^2,8^

1. Department of Water Resources and Environmental Engineering, Tamkang University, No.151, Yingzhuan Rd., Tamsui Dist., New Taipei City 251301, Taiwan.
2. School of Natural Resources and the Environment, The University of Arizona, 1064 East Lowell Street, Tucson, AZ 85721, USA.
3. Department of Geography, Florida State University, 113 Collegiate Loop, PO Box 3062190, Tallahassee, FL, USA.
4. US Geological Survey, Geosciences and Environmental Change Science Center, P.O. Box 25046, MS-980, Denver, CO 80225-0046, USA; [jediffendorfer@usgs.gov](mailto:jediffendorfer@usgs.gov). 303-236-5369
5. US Geological Survey, Upper Midwest Environmental Sciences Center, 2630 Fanta Reed Road, La Crosse, WI 54603, USA.
6. Ecology & Evolutionary Biology Department, The University of Tennessee, 569 Dabney Hall1416 Circle Dr, TN 37996, USA.
7. Institute of Ecology, National Autonomous University of Mexico, University City, Coyoacán, 04510 Mexico City, CDMX, Mexico.
8. Udall Center for Studies in Public Policy, The University of Arizona, 803 E 1st Street, Tucson, AZ 85719, USA.

Disclaimer

Any use of trade, firm, or product names is for descriptive purposes only and does not imply endorsement by the U.S. Government.

**
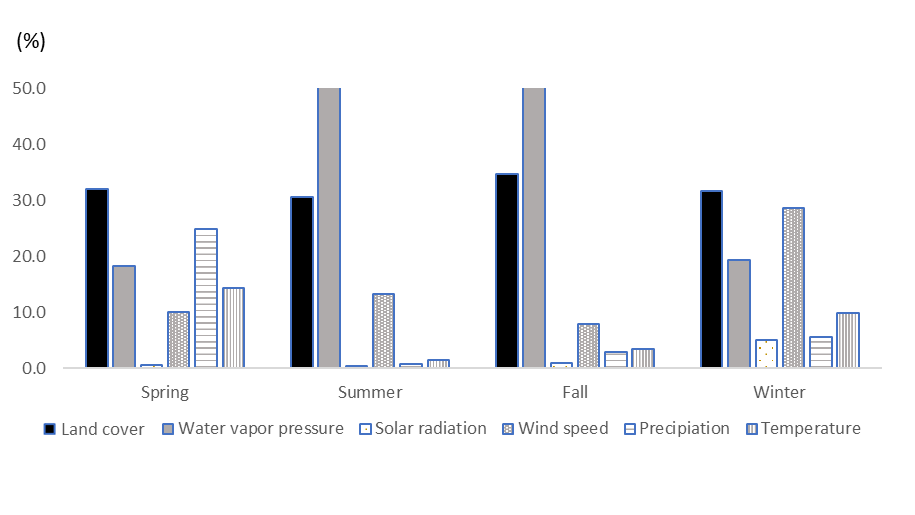
**

**Fig S1.** Graphic representing environmental variable importance (percent contribution/ relative importance) of *T.b. mexicana* for the Maxent distribution models in different seasons.

**Table S1.** Data sources used in the analyses.

| Dataset | Spatial Resolution | Source | URL |
| --- | --- | --- | --- |
| Bat Distribution Modelling | | |  |
| Bat locations | N/A | GBIF | https://www.gbif.org/ |
| Bat Roosts | N/A | Wiederholt et al. 2013 | https://esajournals.onlinelibrary.wiley.com/doi/full /10.1890/ES13-00023.1 |
| Climate variables | 30 seconds | Worldclim2 | http://www.worldclim.com/version2 |
| Land cover | 250 m | NALCMS | http://www.cec.org/north-american-land-change-monitoring-system/ |
| Wind turbines | N/A | USGS | https://eerscmap.usgs.gov/uswtdb |
| Wind turbine suitability modelling | | |  |
| Wind Power class | 2 km | NREL | https://www.nrel.gov/gis/assets/images/us-wind-data.zip |
| Land Cover | 30 x 30 m | USGS | https://www.sciencebase.gov/catalog/item/631405b7d34e36012efa2f9d |
| Human Population Density | County | U.S. Census bureau | https://www.census.gov/programs-surveys/acs/data.html |
| Transmission line(km) | N/A | U.S. DHS | https://hifld-geoplatform.opendata.arcgis.com/ |
| Major roads | N/A | U.S. Census bureau | https://www.census.gov/geographies/mapping-files/time-series/geo/tiger-line-file.html |

**Table S2.** Wind turbine suitability model rating scheme for the variables used in the model. Adapted from Miller and Li (2014). “Rangeland” is the combination of all grassland and shrubland classes in NALCMS. NREL, National Renewable Energy Laboratory

| Suitability  Score for wind turbines | Slope  (Degrees) | NREL Wind  Power Class | Land cover | Human Population Density (people/sq. mile) | Distance to transmission line (km) | Distance to major road  (km) |
| --- | --- | --- | --- | --- | --- | --- |
| High (4) | 0-7 | >4 | Cropland/  Rangeland | 0-25 | <5 | <1 |
| Medium(3) | 7-16 | 4 | NA | 25-50 | 5-10 | 1-2.5 |
| Low(2) | 16-30 | 3 | Barren | 50-100 | 10-15 | 2.5-5 |
| Lowest(1) | 30-40 | 2 | NA | 100-150 | 15-20 | 5-10 |
| Unsuitable(0) | >40 | 1 | NA | >150 | >20 | >10 |

**Table S3.** Suitable areas based on Maxent estimated omission rates (the portion of presence points that were misclassified as nonpresence) for *T.b. mexicana* in different seasons in in the southwestern U.S. and Mexico. (“unsuitable areas: pixel lower than 10% training omission rate , low-suitability areas: pixel training omission rate between 10%-50%, high-suitability areas: pixel > 50% training omission rate)

|  | Unsuitable areas  (km^2^) | Low suitability areas  (km^2^) | High suitability areas  *(*km^2^) |  |
| --- | --- | --- | --- | --- |
| Spring | 2,216,608 | 2,055,255 | 638,507 |  |
| Summer | 1,197,924 | 2,483,756 | 1,229,980 |  |
| Fall | 1,644,259 | 2,272,718 | 996,262 |  |
| Winter | 2,702,733 | 1,791,441 | 415,491 |  |
